# *Pythium* species from rice roots differ in virulence, host colonization and nutritional profile

**DOI:** 10.1186/1471-2229-13-203

**Published:** 2013-12-05

**Authors:** Evelien Van Buyten, Monica Höfte

**Affiliations:** 1 Laboratory of Phytopathology, Department of Crop Protection, Faculty of Bioscience Engineering, Ghent University, Coupure Links 653, B-9000 Ghent, Belgium

**Keywords:** *Pythium*, *Oryza sativa L*, Root-oomycete interaction, Histopathology, Jasmonic acid (JA), Phenoarray, Amino acids

## Abstract

**Background:**

Progressive yield decline in Philippine aerobic rice fields has been recently associated with three closely related *Pythium* spp., *P. arrhenomanes*, *P. graminicola* and *P. inflatum*. To understand their differential virulence towards rice seedlings, we conducted a comparative survey in which three isolates each of *P. arrhenomanes*, *P. graminicola* and *P. inflatum* were selected to investigate host colonization, host responses and carbon utilization profiles using histopathological analyses, phenoarrays, DNA quantifications and gene expression studies.

**Results:**

The isolate of the most virulent species, *P. arrhenomanes*, quickly colonized the outer and inner root tissues of rice seedlings, including the xylem, by which it possibly blocked the water transport and induced severe stunting, wilting and seedling death. The lower virulence of the tested *P. graminicola* and *P. inflatum* isolates seemed to be reflected in slower colonization processes, limited invasion of the vascular stele and less systemic spread, in which cell wall fortification appeared to play a role. Progressive hyphal invasions triggered the production of reactive oxygen species (ROS) and phenolic compounds, which was the strongest for the *P. arrhenomanes* isolate and was delayed or much weaker upon inoculation with the *P. inflatum* isolate. The necrosis marker *OsJamyb* seemed faster and stronger induced by the most virulent isolates. Although the isolate of *P. inflatum* was nutritionally the most versatile, the most virulent *Pythium* isolate appeared physiologically more adapted to its host, evidenced by its broad amino acid utilization profile, including D-amino acids, L-threonine and hydroxyl-L-proline. The latter two compounds have been implicated in plant defense and their use by *P. arrhenomanes* could therefore represent a part of its virulence strategy.

**Conclusions:**

This study illustrates that the differential virulence of rice-pathogenic *P. arrhenomanes, P. graminicola* and *P. inflatum* isolates is related to their root colonization capacity, the intensity of induced root responses and their ability to utilize amino acids in their colonization niche. Accordingly, this paper presents important knowledge concerning rice root infections by oomycetes, which could be helpful to further disentangle virulence tactics of soil-borne pathogens.

## Background

*Pythium* species are ubiquitous soil-borne oomycetes that rank from opportunistic up to highly virulent pathogens on many plant species. They mainly infect young plant tissues and cause pre- and post-emergence damping off or reduce the vigor and growth of surviving seedlings. Besides, they infect mature plant roots, resulting in severe necrosis and stunting [1]. Several important graminaceous crops, including maize, wheat, rice, sugarcane, barley, sorghum and turf grasses have been mentioned to suffer from *Pythium* attacks [2]. Recently, *P. arrhenomanes*, *P. graminicola* and *P. inflatum* were associated with progressive yield decline in Philippine aerobic rice fields [3]. These closely related *Pythium* spp. exhibited a varying degree of virulence towards aerobic rice seedlings, among which *P. arrhenomanes* was the most virulent and inflicted a strong pre- and post-emergence damping-off, and stunting of rice shoots, while *P. graminicola* was less virulent and *P. inflatum* is nonpathogenic *in vivo*.

Few histopathological studies have monitored the infection process of *Pythium* spp. in monocot roots. We lately surveyed the interaction of *P. graminicola* with rice seedling roots [4] and revealed that this pathogen quickly invaded the rice rhizodermis via penetration hyphae, after which it generated a dense intracellular network in the inner root tissues and ultimately triggered necrosis. Modjehi et al. (1991) [5] investigated the infection of wheat roots by *P. arrhenomanes* and in this study, the oomycete appeared to penetrate wheat roots via appressoria-like structures. This pathogen also intracellularly invaded the root cortex and, to a lesser extent, the stele, which eventually resulted in severe cortical cell collapse and strong browning of wheat roots. In addition, the interaction between *P. arrhenomanes* and corn has been analyzed [6], and demonstrated that *P. arrhenomanes* developed two different types of hyphae during its colonization of corn roots. To our knowledge, studies on the interaction of rice roots with *P. arrhenomanes* or *P. inflatum* are currently lacking. Furthermore, comparative histopathological analyses with highly and weakly virulent *Pythium* spp. on monocot roots have never been executed.

Therefore, we explored the rice root colonization processes of one isolate each of *P. arrhenomanes*, *P. graminicola* and *P. inflatum* from diseased aerobic rice fields in the Philippines. *In vitro* infection trials allowed the accurate evaluation of rice root and shoot development upon *Pythium* inoculation, and histological and molecular techniques were used to unravel qualitative and quantitative differences among the infection processes of these three *Pythium* isolates*.* Using similar techniques, rice root responses to *Pythium* were investigated over time. In addition, phenoarrays were carried out to reveal the nutritional needs of the three oomycetes and to identify carbon-utilization patterns related to a higher virulence. Our research discovered clear differences in the root colonization capacity and nutritional profiles of rice-infecting *P. arrhenomanes, P. graminicola* and *P. inflatum* isolates.

## Results

### Macroscopic symptoms on rice seedlings upon *Pythium* infection

**Figure 1:**
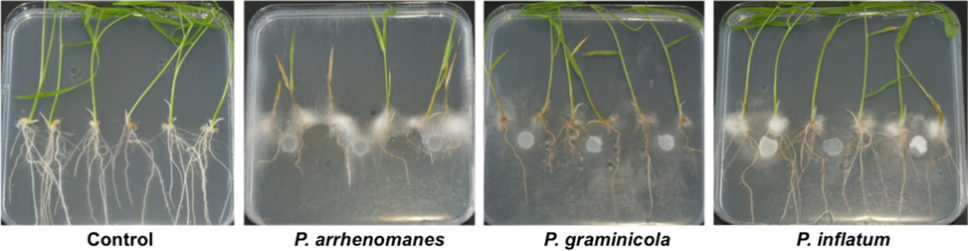
**Disease symptoms on rice seedlings 10 days after *Pythium *inoculation.** Clear differences in stunting and wilting were noticed among *P. arrhenomanes-*, *P. graminicola-* and *P. inflatum-*inoculated rice seedlings. Rice seeds were germinated on Gamborg B5 medium and three days post imbibition, seedlings were inoculated with mycelial plugs. Three replicate plates were evaluated per treatment. Pictures are representative for all replicates.

Rice seedlings (cv. Nipponbare) were cultured on Gamborg B5 (GB5) agar plates to study the effect of *P. arrhenomanes* PT 60, *P. graminicola* PB912 132 and *P. inflatum* PT 52 on root and shoot development. Macroscopic evaluation of *Pythium*-inoculated rice seedlings illustrated the intense colonization of rice tissues by the *P. arrhenomanes* isolate (Figure 1). Both rice seed and root surfaces became massively covered with aerial mycelium by 2 days post inoculation (dpi). When the infection proceeded, the mycelium concentrated primarily on and in the vicinity of the rice seeds. Similarly, a dense white mycelium appeared on the seeds of *P. inflatum* PT 52-inoculated cultures by 2 dpi. The superficial colonization was, however, less pronounced in this case. Hyphae of *P. graminicola* PB912 132 grew well in the medium adjacent to the rice seeds, but rice seedling surfaces were never heavily colonized. When nutrients were eliminated from the medium, we still observed a stimulated hyphal growth near the rice seeds (data not shown). This growth stimulation was probably due to seed exudation. When the *Pythium* isolates were cultured in seed exudates alone, an increased hyphal growth was indeed observed (Additional file 1: Figure S1).

Evaluation of the *in vitro* cultures at 10 dpi revealed that rice seedlings were very susceptible to *P. arrhenomanes* PT 60 and *P. graminicola* PB912 132 infection (Figure 1, Table 1). The most virulent isolate *P. arrhenomanes* PT 60 inhibited crown root and lateral root formation. Besides, primary root lengths were significantly reduced by 63% compared to the non-inoculated control (*P* ≤ 0.05). Necrosis was visible as typical brown discolorations on the upper part of the primary roots. When shoot growth was monitored, a significant stunting of 61% relative to the control became evident (*P* ≤ 0.05). Furthermore, 72% of all inoculated rice seedlings exhibited clear wilting symptoms, visible as yellowing or browning of culms and/or leaves. The high virulence level of *P. arrhenomanes* PT 60 also resulted in a significant seedling death of 28% (*P* ≤ 0.05), i.e. the percentage of plantlets with shoot disease severity score 5.

**Table 1:** ***Pythium*-induced disease symptoms on rice seedlings at 10 dpi**

**Species**	Shoot length (cm)^*^	Root length (cm)^*^	DSI%^**^	Dead seedlings %^***^
Control	16.07 (0.34) **a**	9.19 (0.43) **a**	3 (1.92) **a**	0 **a**
*P. arrhenomanes*	6.24 (0.62) **b**	3.42 (0.15) **b**	59 (7.22) **b**	28 (11.11) **b**
*P. graminicola*	10.26 (0.57) **c**	4.74 (0.23) **c**	32 (1.00) **c**	0 **a**
*P. inflatum*	17.97 (0.42) **a**	9.06 (0.50) **a**	23 (0) **d**	0 **a**

^*^Mean shoot and root length data (n = 18).

^**^Average disease severity indices (DSI) (n = 3) were calculated using the disease severity scale (see “Methods”). Indices vary from 0% (healthy shoots) up to 100% (dead shoots) and illustrate the degree of yellowing or browning in culms and leaves in parallel with shoot stunting. Eighteen plantlets were individually scored for each treatment.

^***^The percentage of dead seedlings displays the mean number of shoots (n = 3) with disease severity score 5.

Statistical analyses were mostly performed by Kruskal-Wallis and Mann–Whitney non-parametric tests in SPSS 21 (SPSS Inc.). Only shoot length data were normally distributed and therefore, analyzed with one-way ANOVA and the Duncan Post-Hoc test. In each column, distinct lower case letters (a,b,c,d) indicate significant differences between the treatments (α = 0.05, *P* ≤ α)*.* Values between brackets are standard errors.

In contrast to the *P. arrhenomanes* isolate, *P. graminicola* PB912 132 never impaired crown root and lateral root formation in rice seedlings. Nonetheless, all root types were highly stunted and exhibited an overall clear brown discoloration. Primary root lengths were significantly shorter (*P* ≤ 0.05), with lengths representing 52% of those of the non-inoculated control. Opposing the strong wilting symptoms on *P. arrhenomanes* PT 60-inoculated rice seedlings, shoots appeared significantly healthier (*P* ≤ 0.05) and survived all upon *P. graminicola* PB912 132 inoculation. Moreover, shoot growth was reduced by 36%, which was significantly less (*P* ≤ 0.05) than the stunting evoked by the most virulent isolate. The impact of *P. inflatum* PT 52 on rice seedling development was rather minor in comparison with the isolates of the other species (Figure 1, Table 1). Except for a curtailed crown root elongation and necrotic patches on primary, crown and lateral roots, no apparent disease symptoms were observed. Maximal root lengths of *P. inflatum* PT 52-inoculated seedlings represented 99% of the control treatment. In addition, shoot stunting was only 7% and appeared not significant (*P* < 0.05). Most shoots were categorized in the lowest disease score, exhibiting only few symptoms of wilting. Disease severity indices were still significantly distinct from the control treatment (*P* ≤ 0.05) indicating that *P. inflatum* is weakly virulent *in vitro*.

### Histological study of the *Pythium* infection process in rice roots

**Figure 2:**
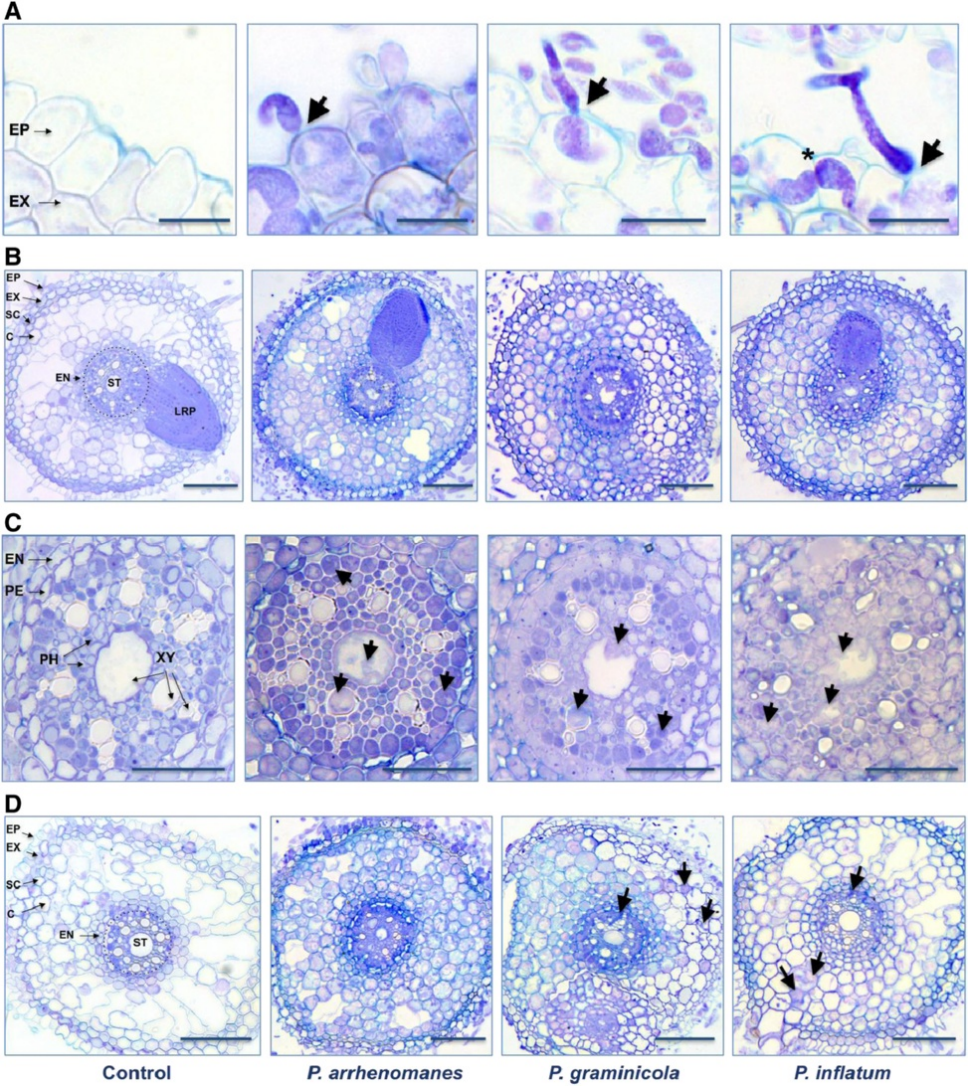
**Histological study of the *Pythium *infection process in rice roots. A**, Direct penetration of the rice rhizodermis by *P. arrhenomanes, P. graminicola* and *P. inflatum*. Arrows indicate sites of hyhal penetration. Bulbous-like *Pythium* hyphae grew intracellularly and became severely constricted right before cell wall passing (*). **B**, Colonization of the inner rice root tissues by *P. arrhenomanes, P. graminicola* and *P. inflatum* in the most heavily infected (upper) parts of the primary root at 27 hpi. **C**, Colonization of the stele by *P. arrhenomanes, P. graminicola* and *P. inflatum* in the most heavily infected parts of the primary root at 27 hpi. Arrows indicate hyphae in the phloem and xylem. **D**, Colonization of the inner root tissues 2 days upon inoculation with *P. arrhenomanes*, *P. graminicola* and *P. inflatum* in the middle part of the primary root. *P. graminicola* and *P. inflatum* hyphae were less abundant in the cortex and vascular tissues (arrows). Abbreviations in the control pictures designate the epidermis (EP), exodermis (EX), sclerenchyma (SC), cortex (C), endodermis (EN), pericycle (PE), stele (ST), phloem (PH), xylem (XY) and lateral root primordium (LRP). Cross-sections (5 μm) from *Pythium*-infected root tissues were stained with trypan blue. Scale bars in A = 20 μm, in B = 100 μm, in C = 50 μm and in D = 100 μm.

To investigate the colonization process of *P. arrhenomanes* PT 60, *P. graminicola* PB912 132 and *P. inflatum* PT 52 inside rice roots, we examined trypan blue-stained root cuttings with bright field microscopy. The three *Pythium* isolates invaded primary roots via direct penetration of epidermal cells (Figure 2A). Swollen hyphae rather than specialized appressoria were used as penetration tools. Immediately after *Pythium* ingress, hyphae differentiated in irregularly inflated structures that headed intracellularly to the walls of neighboring cells. Right before cell wall crossing, hyphae became dramatically constricted (Figure 2A). The same strategy was likely adopted during the further spread of *Pythium* hyphae in the cortex and vascular tissue.

However, the isolates of *P. arrhenomanes*, *P. graminicola* and *P. inflatum* differed in the extent to which inner root tissues were colonized. In the most heavily infected parts of the primary root (the upper 1.5 cm), the three *Pythium* isolates strongly colonized the cortex, endodermis and stele within 27 hpi (Figure 2B). A more detailed view onto the stele unraveled the numerous hyphae that were present in the phloem and xylem by that time (Figure 2C). Histological studies on the middle part of the primary root generated similar results for the *P. arrhenomanes* PT 60 colonization process, while *P. graminicola* PB912 132 hyphae were less abundant in the cortex and vascular tissue (Figure 2D). Besides, the latter pathogen did not invade xylem cells in the majority of the examined cross-sections. In *P. inflatum* PT 52-inoculated root samples, the occurrence of a dense hyphal network was less frequent compared to *P. arrhenomanes* PT 60 and *P. graminicola* PB912 132-inoculated root samples. In addition, the colonization of the root stele was often limited to the outer cell layers.

### Quantification of *Pythium* DNA in rice roots

It has been stated that the combination of qualitative and quantitative techniques allows more accurate time-course monitoring of microbial infection processes in plant roots [7]. Therefore, a DNA-based quantification method was used to further survey the colonization of rice seedling roots by *P. arrhenomanes* PT 60*, P. graminicola* PB912 132 and *P. inflatum* PT 52. Quantitative Real-Time PCR (qPCR) on non-surface-sterilized root samples illustrated that *P. arrhenomanes* PT 60 quickly and massively colonized the entire rice root system (Table 2A). In a first experiment (Exp 1), DNA quantities of the tested *P. arrhenomanes* isolate represented 8.42% of the total DNA extract at 1 dpi, which strongly increased up to 49.2% and 59% by 2 and 3 dpi, respectively. Quantities of *P. graminicola* PB912 132 DNA were much lower and maintained more or less the same level over time (i.e. 5-6%). The concentration of *P. inflatum* PT 52 DNA on and in rice seedlings roots elevated from 1.39% at 1 dpi up to 26.4% at 2 dpi, after which it diminished down to 15.8% by 3 dpi.

**Table 2:** ***In planta *** **quantification of *Pythium *** **DNA (pg/ng total DNA) using qPCR**

	P. arrhenomanes*		P. graminicola*		P. inflatum*	
	**Exp 1**	**Exp 2**	**Exp 1**	**Exp 2**	**Exp 1**	**Exp 2**
**A**						
**1 dpi**	84.24 (0.40)	179.26 (17.85)	57.76 (4.70)	179.64 (8.94)	13.88 (0.64)	2.89 (0.27)
**2 dpi**	492.00 (28.28)	291.42 (19.37)	47.28 (0.85)	142.00 (30.60)	264.10 (03.65)	113.44 (2.09)
**3 dpi**	590.00 (36.77)	405.32 (82.99)	58.40 (1.58)	51.14 (10.78)	157.70 (25.37)	196.16 (6.79)
**B**						
**20 hpi**	1.246 (0.141)	2.162 (2.437)	0.330 (0.027)	0.031 (0.001)	0.010 (0.006)	0.003 (0.001)
**28 hpi**	8.434 (0.240)	8.782 (0.167)	8.958 (0.325)	1.128 (0.082)	0.966 (0.040)	0.108 (0.006)
**73 hpi**	47.680 (4.299)	4.564 (0.238)	55.320 (6.505)	0.332 (0.040)	0.857 (0.157)	0.019 (0.001)

*In each experiment (Exp 1 and 2) 12 rice seedlings were pooled per treatment at various times. The presented data are means of two technical replicates (n = 2). Values between brackets represent standard deviations.

**A,** Concentration of *Pythium* DNA in non-surface sterilized rice seedlings roots. Seeds were germinated on Gamborg B5 medium and inoculated at three days post imbibition. Root samples were collected in two different experiments at various times upon inoculation. **B,** Concentration of *Pythium* DNA in surface sterilized rice seedling roots. Seeds were germinated on plant agar-medium and inoculated at three days post imbibition. Rice roots were collected in two different experiments at various times upon inoculation and were surface-sterilized prior to processing.

A second experiment (Exp 2) generated similar increasing trends for the colonization process of the *P. arrhenomanes* isolate. In *P. graminicola* PB912 132- and *P. inflatum* PT 52-inoculated roots, consistent DNA concentrations were measured at 3 dpi (i.e. 5% and 19.6%, respectively), but during the first days, the quantities were respectively higher and lower compared to the first experiment.

qPCR on surface-sterilized root samples enabled the quantification of *Pythium* DNA inside the rice root system. Once more, the concentration of *P. arrhenomanes* PT 60 DNA clearly elevated over time (Table 2B), with quantities of 0.13% at 20 hpi that multiplied up to 0.84% at 28 hpi and 4.77% at 73 hpi (Exp 1). In contrast, the concentration of *P. graminicola* PB912 132 DNA inside the rice seedling root system was much lower at 20 hpi. Nonetheless, by 73 hpi, DNA quantities of the *P. arrhenomanes* and *P. graminicola* isolates attained equal amounts. The share of *P. inflatum* PT 52 DNA in the total DNA extract was very low during the first 20 h of the infection process. From 28 hpi on, DNA quantities slightly elevated up to approximately 0.1%, but remained much lower than those of the other species. Despite the overall lower infection level in our second experiment, we could observe similar colonization trends for the three *Pythium* isolates. Taken together, these data imply that the tested *P. arrhenomanes* isolate is a better and faster colonizer of rice root surfaces and inner tissues than the screened *P. graminicola* and *P. inflatum* isolates. Furthermore, they demonstrate that the isolate of the weakly virulent species *P. inflatum* mainly colonizes rice root surfaces, while the isolate of *P. graminicola* is less efficient in its superficial spread.

### Rice root responses to *Pythium* spp

#### Reactive oxygen species (ROS)

The accumulation of hydrogen peroxide (H_2_O_2_) in *Pythium*-inoculated rice seedling roots was visualized with bright field microscopy and an endogenous peroxidase-dependent staining procedure using 3,3’-diaminobenzidine (DAB).

**Figure 3:**
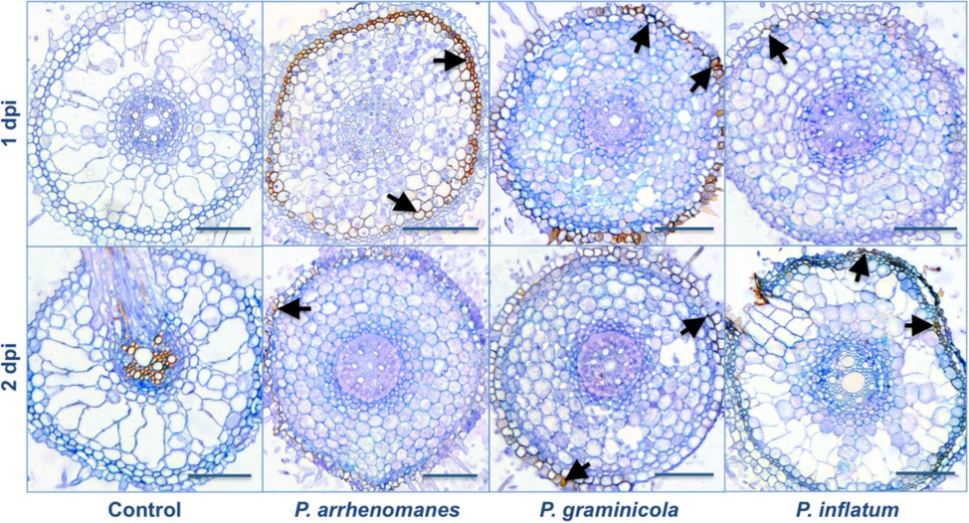
**H**_**2**_**0**_**2 **_**production in *Pythium*-inoculated rice roots at 1 and 2 dpi.** Arrows indicate reddish-brown DAB precipitates. Samples were taken from the most heavily infected parts of the primary root (upper 1.5 cm) and infiltrated with DAB. After cross sectioning, samples (5 μm) were stained with trypan blue. Scale bars = 100 μm.

When the most heavily infested (upper) parts of the primary roots were analyzed, we detected reddish-brown DAB precipitates in the outer cell layers (Figure 3, black arrows). At 1 dpi, H_2_O_2_ production appeared the strongest in *P. arrhenomanes* PT 60-inoculated seedling roots, where they accumulated in the sclerenchyma and some cortical cells. By 2 dpi, these DAB precipitates became less visible. In *P. graminicola* PB912 132-inoculated seedlings, DAB concentrated in the epidermis and exodermis of the roots, and this H_2_O_2_ production also diminished by 2 dpi. On the contrary, DAB precipitates were barely noticeable during the first 24 h of the *P. inflatum* PT 52-rice interaction. By 2 dpi, the H_2_O_2_ production slightly increased in the epidermis, exodermis and sclerenchyma of seedling roots, but overall rice roots seemed to respond less upon inoculation with this isolate. DAB accumulation did not occur in control roots, with the exception of the vascular tissue and aerenchym.

**Figure 4:**
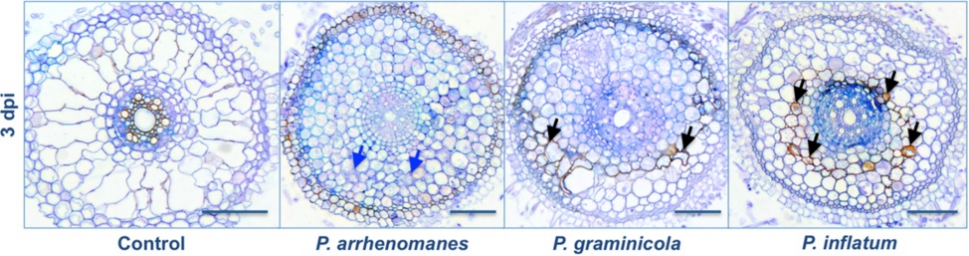
**H**_**2**_**0**_**2**_** production in *Pythium*-inoculated rice roots at 3 dpi.** Black arrows indicate reddish-brown DAB precipitates, which seemed to slow down hyphal spread in the cortex of *P. graminicola*- and *P. inflatum*-infected rice roots. Blue arrows indicate an area of cell collapse in *P. arrhenomanes*-inoculated roots. Samples were taken in the middle of the primary root and infiltrated with DAB. After cross sectioning, samples (5 μm) were stained with trypan blue. Scale bars = 100 μm.

When the middle part of the primary root was examined, we also observed DAB accumulation in the outer and inner cortex of *P. inflatum* PT 52- and *P. graminicola* PB912 132-inoculated seedlings at 3 dpi (Figure 4, black arrows). In both cases, hyphal proliferation in a part of the root cortex was hampered and colonization was slowed down. In *P. arrhenomanes* PT 60-inoculated seedlings, cortical cells seemed to collapse at this stage of the infection (Figure 4, blue arrows).

#### Phenolic compounds

The accumulation of phenolic compounds in *Pythium*-inoculated rice seedling roots was visualized as an orange-brown autofluorescence by UV-excitation of calcofluor white M2R-stained root cuttings.

**Figure 5:**
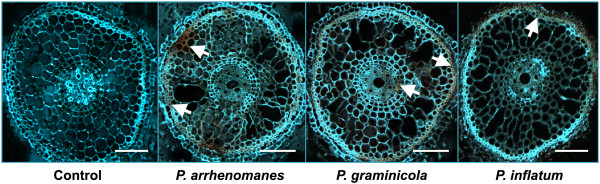
**Accumulation of phenolic compounds in *Pythium*-inoculated rice roots at 2 dpi.** White arrows indicate the orange-colored autofluorescence upon *Pythium* infection. Cross-sections (5 μm) from *Pythium*-infected root tissues were stained with Calcofluor white M2R and excited with UV. Scale bars = 100 μm.

The autofluorescence was visible from 2 dpi on (Figure 5, white arrows) and was the strongest in *P. arrhenomanes* PT 60-inoculated rice seedling roots, where it was emitted from parts of the outer root cortex and the sclerenchyma. In *P. graminicola* PB912 132-inoculated root cuttings, phenolic compounds were primarily visible in the sclerenchyma and little autofluorescence was emitted from the vascular tissue. Similarly, we detected autofluorescence in the sclerenchyma of *P. inflatum* PT 52-inoculated rice roots, but once more, the host responded the weakest to this isolate. No autofluorescence was detected in control roots.

#### Expression of the necrosis marker and JA-responsive gene OsJAmyb

**Figure 6:**
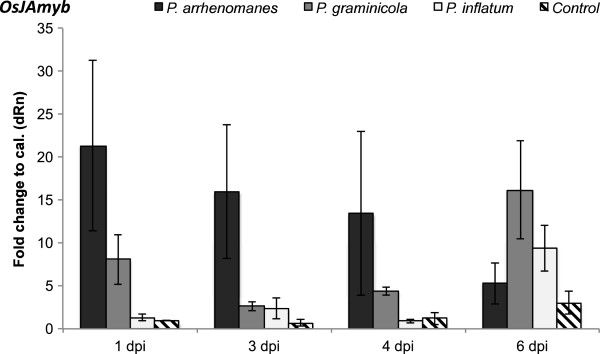
**Transcription of *OsJAmyb *in *Pythium*-inoculated rice roots at 1, 3, 4 and 6 dpi.** Transcript levels were normalized against the internal reference actin and relatively expressed to the control at 1dpi. Data are means of three different experiments (n = 3), each experiment representing a pooled sample from at least six rice plantlets. Error bars display the standard error values.

To compare the degree of induced necrosis among *P. arrhenomanes* PT 60-, *P. graminicola* PB912 132- and *P. inflatum* PT 52-inoculated root systems, the expression of *OsJAmyb* was analyzed by qPCR analysis. This JA- and pathogen-inducible MYB transcription factor has been identified as a necrosis marker and is mainly expressed in plant tissues prior to cell death [8]. Our gene expression analysis revealed that the three *Pythium* isolates induced necrosis in rice seedling roots (Figure 6). *P. arrhenomanes* PT 60 showed to strongly trigger necrosis at 1, 3 and 4 dpi, when a respective 21.3-fold, 16-fold and 13.4-fold induction in *OsJAmyb* transcription was measured. By 6 dpi, the transcription decreased down to 5.3-fold and approximated the expression level in the control. In *P. graminicola* PB912 132-inoculated root tissues we noted a lower induction of *OsJAmyb* during the first days of the infection. By 6 dpi, the expression of the necrosis marker strongly elevated up to 16.2-fold and seemed to exceed the level in *P. arrhenomanes* PT 60-inoculated roots. Necrosis was barely triggered during the first days upon inoculation with the *P. inflatum* isolate. Only at 6 dpi, mRNA levels strongly elevated (9.4-fold).

*Isolates of P. arrhenomanes and P. graminicola are nutritionally less versatile than that of P. inflatum*

**Figure 7:**
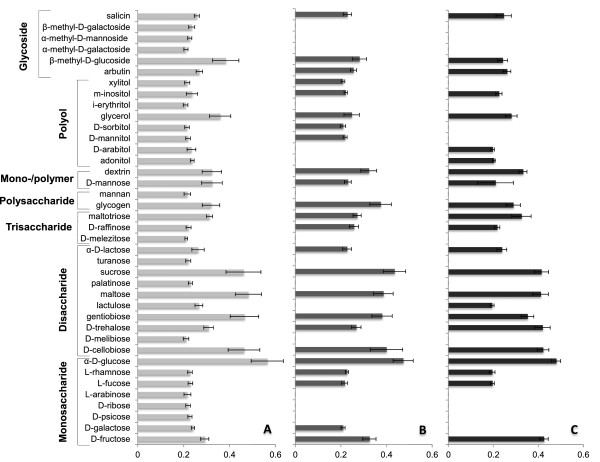
**Carbohydrates and derivatives metabolized during *in vitro *growth of rice-infecting *Pythium *spp. A, B** and **C** represent the profiles of *P. inflatum*, *P. arrhenomanes* and *P. graminicola*, respectively. Turbidimetrical measurements were executed 24 hpi. The experiment consisted of three replicate plates per treatment and was repeated in time (n ≥ 6). Only carbon sources with OD-values significantly different from the control-treatment are presented. Statistical analyses were performed using the Kruskal-Wallis non-parametric test in SPSS 21 (α = 0.05, *P* ≤ α).

**Figure 8:**
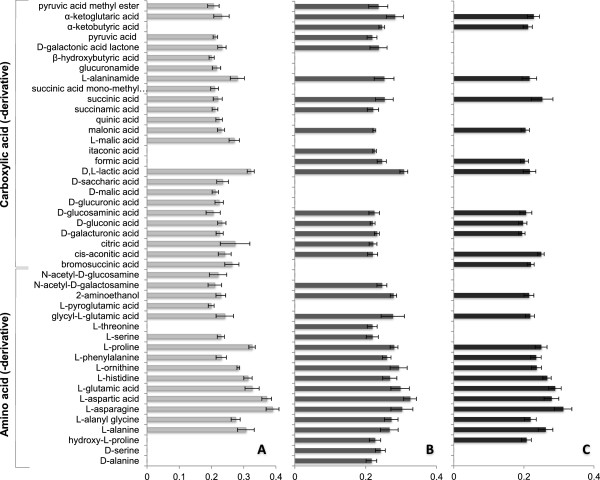
**Amino acids, carboxylic acids and derivatives metabolized during *in vitro *growth of rice-infecting *Pythium *spp. A, B** and **C** represent the profiles of *P. inflatum*, *P. arrhenomanes* and *P. graminicola*, respectively. Turbidimetrical measurements were executed 24 hpi. The experiment consisted of three replicate plates per treatment and was repeated in time (n ≥ 6). Only carbon sources with OD-values significantly different from the control-treatment are presented. Statistical analyses were performed using the Kruskal-Wallis non-parametric test in SPSS 21 (α = 0.05, *P* ≤ α).

The growth of one isolate each of *P. arrhenomanes*, *P. graminicola* and *P. inflatum* on various carbon sources was evaluated using phenoarrays. This analysis revealed the ability of *P. inflatum* isolate PT 52 to use a broad range of carbohydrates, amino acids, carboxylic acids and derivatives (Figure 7 and 8).

The *P. inflatum* isolate used 80% of all mono-, di-, tri- and polysaccharides, sugar monomers/polymers, glycosides and polyols supplemented to the MicroPlates, while this was only 49% and 47% for *P. arrhenomanes* PT 60 and *P. graminicola* PB912 132, respectively (Figure 7; Additional file 2: Table S1, Additional file 3: Table S2). The latter isolates did not metabolize glycosides of galactose and mannose, while all glycosides stimulated the growth of *P. inflatum* PT 52. Furthermore, only 56% and 44% of the tested polyols were utilized by *P. arrhenomanes* PT 60 and *P. graminicola* PB912 132, respectively, whereas this was 89% in the case of *P. inflatum* PT 52. It was interesting to see that the tested isolates of *P. arrhenomanes* and *P. graminicola* grew on different polyols beside glycerol and m-inositol. Compared to the *P. inflatum* isolate, they were also less efficient in their use of several saccharides, in particularly monosaccharides. Nevertheless, the overall highest absorbance values (OD < 0.35) were measured in wells pre-filled with the disaccharides sucrose, maltose, gentiobiose and D-cellobiose, and the monosaccharide α-D-glucose. Turbidities in wells inoculated with *P. inflatum* PT52 were the highest for these compounds, supporting its better growth on carbohydrates compared to the other species. Besides, the *P. graminicola* and *P. arrhenomanes* isolates grew the best on D-fructose (monosaccharide) and D-trehalose (disaccharide), and glycogen (polysaccharide), respectively. *P. arrhenomanes* PT 60 and *P. inflatum* PT 52 shared the ability to use D-galactose, while the isolates of *P. inflatum* and *P. graminicola,* but not *P. arrhenomanes*, were able to use lactulose.

When exploring the growth of the three *Pythium* isolates on amino acids, carboxylic acids and their derivatives, it became clear that *P. inflatum* PT 52 and *P. arrhenomanes* PT 60 could use respectively 68% and 61% of these compounds, while this was only 43% for *P. graminicola* PB912 132 (Figure 8; Additional file 2: Table S1, Additional file 3: Table S2). The screened isolate of *P. inflatum* exhibited the broadest carboxylic acid-profile and was the only isolate that could utilize quinic acid, D,L-malic acid, D-saccharic acid, D-glucuronic acid and β-hydroxybutyric acid. Nevertheless, it was not able to metabolize two particular carboxylic acids, i.e. formic acid and α-ketobutyric acid, which intriguingly showed to stimulate the growth of the more virulent isolates. Aside from these findings, *P. arrhenomanes* PT 60 appeared unique in its ability to use itaconic acid, while no carboxylic acids or derivatives seemed specific for *P. graminicola* PB912 132 (Table 3). We also identified certain carboxylic acids that enhanced the growth of either the *P. arrhenomanes* and *P. inflatum* isolates or the *P. graminicola* and *P. inflatum* isolates (Table 3).

**Table 3:** **Carbon sources specific to *P. arrhenomanes *** **and/or *P. graminicola, *** **and/or shared with *P. inflatum***

**Carbon source**	P. arrhenomanes*	P. graminicola*	P. inflatum*
D-alanine	+	-	-
D-serine	+	-	-
Itaconic acid	+	-	-
L-threonine	+	-	-
Formic acid	+	+	-
Hydroxy-L-proline	+	+	-
α-ketobutyric acid	+	+	-
Citric acid	+	-	+
D-galactose	+	-	+
D-mannitol	+	-	+
D-sorbitol	+	-	+
L-serine	+	-	+
N-acetyl-D-galactosamine	+	-	+
Pyruvic acid	+	-	+
Pyruvic acid methyl ester	+	-	+
Succinamic acid	+	-	+
Xylitol	+	-	+
Adonitol	-	+	+
Bromosuccinic acid	-	+	+
D-arabitol	-	+	+
Lactulose	-	+	+

* + or - indicate if the *Pythium* spp. could respectively grow or not on the according carbon sources. Statistical analyses were performed using Kruskal-Wallis non-parametric tests in SPSS 21 (SPSS Inc.) (α = 0.05, *P* ≤ α).

The amino acid-profiles of all tested *Pythium* isolates included L-alanine, L-alanyl-glycine, L-asparagine, L-aspartic acid, L-glutamic acid, L-histidine, L-ornithine, L-phenylalanine and L-proline. However, L-threonine, D-serine and D-alanine were exclusively used by *P. arrhenomanes* PT60 and hence, it exhibited the broadest amino acid profile among the three *Pythium* isolates (Figure 8). *P. inflatum* PT 52 and *P. arrhenomanes* PT 60 could both utilize L-serine, while *P. graminicola* PB912 132 could not. Interestingly, the use of hydroxy-L-proline seemed specific for the more virulent *Pythium* isolates and none of the three isolates showed growth on L-leucine (Additional file 3: Table S2).

The three *Pythium* isolates also responded differently to certain other carbon sources (Additional file 2: Table S1). The nucleosides thymidine, uridine and inosine, triggered the growth of *P. arrhenomanes* PT 60, while *P. inflatum* PT 52 seemed to be the only tested isolate that could grow on putrescine.

## Discussion

In this study, one isolate each of *P. arrhenomanes*, *P. graminicola* and *P. inflatum* from diseased aerobic rice fields [3] were selected for a detailed *in vitro* analysis of their host infection process and the consequent disease development. *In vitro* inoculation of rice seedling roots revealed that the *P. arrhenomanes* isolate was clearly more virulent than the isolates of *P. graminicola* and *P. inflatum*. This pathogen inhibited crown and lateral root development, induced severe wilting and caused strong stunting of rice roots and shoots, whereas the disease symptoms induced by the *P. graminicola* isolate were less pronounced and the *P. inflatum* isolate exerted only minor effects on rice seedling development.

Histopathological studies revealed that the three *Pythium* isolates invaded rice seedling roots using penetration hyphae. Once inside the root tissue, *Pythium* hyphae became irregularly inflated and grew mainly intracellular. Cell wall crossing occurred by means of constricted hyphae, which probably migrated through the plasmodesmata and hence, avoided visible cell wall damage. Consistent findings were presented for *Pythium* root infections in other monocots [5,6,9]. However, specialized appressoria-like structures instead of simple hyphae enabled rhizodermal penetration in these cases [5]. After pathogen ingress, we found that the *P. arrhenomanes*, *P. graminicola* and *P. inflatum* isolates massively parasitized the inner rice root tissues by hyphal ramification, resulting in the complete filling of cortical and endodermal cells within 27 hpi. Comparably, dense hyphal networks were noted upon infection of *Arabidopsis* plantlets with *P. irregulare*[10].

In parallel with their varying virulence, we showed that the three *Pythium* isolates differed in the extent to which systemic tissues were colonized. The isolate of *P. arrhenomanes* quickly colonized the entire primary root, efficiently spreading from the primary infection site to other parts.

Besides, hyphae abundantly invaded the xylem and accordingly, might have blocked the water transport to the shoot, partly explaining the severe wilting and frequent death of rice seedlings upon inoculation with this isolate. Such parasitic features are common for true vascular pathogens like *Fusarium oxysporum* and *Verticillium* spp., where hyphae invade the plant root stele before the endodermis is suberized [11]. In case of *Pythium* infections, however, hyphal blocking of xylem has not often been reported. Extensive invasion of the vascular stele has only been described for *P. irregulare* on Arabidopsis [10], *P. tracheiphilum* on lettuce [12], *P. sylvaticum* and *P. dissotocum* on strawberry [13] and *P. ultimum* on cucumber [14]. In the latter case, xylem vessels were also occluded by the production of plant defense-related tyloses that attempted to limit hyphal spread.

The systemic spread of the *P. inflatum* and, to a lesser extent, the *P. graminicola* isolate, in the cortex and stele of rice seedling roots, was more limited than that of the *P. arrhenomanes* isolate. Especially xylem vessels appeared less colonized by the *P. graminicola* isolate, whereas the growth of the *P. inflatum* isolate was often limited to few phloem cells, possibly underlying the lower degree of wilting and stunting upon inoculation with these pathogens. Our qPCR analysis confirmed that the *P. arrhenomanes* isolate was the best colonizer of the rice seedling root system, followed by the isolates of *P. graminicola* and *P. inflatum*, with the latter barely spreading in the inner tissues. Such a positive correlation between *Pythium* root colonization capacity and virulence is not always to be expected. In contrast to our results, Modjehi et al. (1991) [5] reported that in wheat roots, most *P. arrhenomanes* hyphae are blocked at the endodermis. In this study, the stele remained unaffected at distance of the primary infection site until the cells died. Moreover, the colonization process of *P. arrhenomanes* proceeded more slowly in this host than in rice roots. Other contrasting findings have been described for *Pythium* group F-tomato root interactions. *Pythium* group F is a minor pathogen that may cause yield losses without producing visible root symptoms [15]. In tomato roots, it is able to colonize all cell types, including the xylem, within 2-3 dpi. However, it has been discovered that most of the xylem-invading hyphae appear as empty ghost cells.

Similar findings have been reported for the biocontrol agent and growth-promoting species *P. oligandrum* in its interaction with tomato roots [16].

The progressive invasions of *Pythium* hyphae in rice seedling roots triggered the production of reactive oxygen species (ROS) and phenolic compounds, which ultimately evoked cell death. ROS are short-lived molecules that interact with proteins, DNA, lipids and carbohydrates in plant cells, and thereby induce tissue damage and cell death [17]. Plants produce scavengers for anti-oxidative protection. However, several biotic and abiotic factors may disturb the balance between ROS and scavengers, and so, evoke an oxidative burst [18]. Necrotrophic pathogens for instance, may trigger the intracellular production of ROS during the killing of host tissues. On the other hand, ROS-production may as well be part of plant defense responses to biotrophic and hemi-biotrophic pathogens [17]. In the current study, production of hydrogen peroxide (H_2_O_2_) was observed in the outer cell layers of *Pythium*-inoculated rice tissues and was stronger for the more virulent species at 1 dpi. Oliver et al. (2009) [19] also noticed ROS production upon *P. irregulare* and *P. debaryanum* infection of moss, but in theses cases, ROS accumulated in the *Pythium*-spreading area, where it preceded cell death. The three *Pythium* isolates invaded the stele of rice seedling roots by 1 dpi, while DAB concentrated far behind the infection front, suggesting that the observed H_2_O_2_ accumulation might be related to immune reactions that arose too late to prevent infection. These likely included cell wall modification events, since *Pythium* spp., like other oomycetes, directly penetrate their host [19] and hydrogen peroxide is involved in the peroxidase-catalyzed cross-linking of cell wall polymers [20]. However, it is also possible that the H_2_O_2_ accumulation in *Pythium*-infected rice roots was indicative for cell death upon nutrient depletion [21]. Such a trailing necrosis has also been described for *P. irregulare*-Arabidopsis interactions [10]. Histological analyses of *P. arrhenomanes*-inoculated rice cultures revealed cell collapse in the root cortex at 3 dpi, which confirmed the occurrence of necrosis.

Cell collapse resulting from *P. arrhenomanes* infections of wheat seedlings has been previously linked with toxin or enzyme production [5]. Toxins or substantial amounts of cell wall degrading enzymes are likely not involved in the *Pythium*-rice interaction, because pathogen invasion was not associated with extensive tissue damage and was not preceded by cell death. In root tissues inoculated with the *P. inflatum* and *P. graminicola* isolates, we also detected a cortex-related ROS production at 3 dpi that slowed down the *Pythium* colonization process, suggesting that cell wall strengthening occurred and that the interaction between rice roots and *P. inflatum* or *P. graminicola* might be less compatible than with *P. arrhenomanes*.

Beside ROS, phenolic compounds fulfill major roles in the response of plants to biotic and abiotic stresses [22]. *Pythium* infections have been mentioned to elicit the accumulation of phenolics [16,19], which can be incorporated into the cell wall during fortification events or may be liberated during cell death [19]. These phenolic accumulations are microscopically visible as autofluorescence upon UV-irradiation [22] or macroscopically as root browning [23]. In the present paper, we detected a similar root browning in *Pythium*-inoculated rice cultures. When inner root tissues were studied, we noted the strongest autofluorescence in the cortex of primary roots inoculated with the *P. arrhenomanes* isolate, while autofluorescence was mainly emitted from the sclerenchyma upon inoculation with the *P. graminicola* isolate and was weakly visible in the outer tissues upon inoculation with the *P. inflatum* isolate. Taken together, these results suggest that rice seedlings might respond more and/or faster to highly virulent than weakly virulent *Pythium* species.

Elevated expression levels of necrosis marker *OsJAmyb* in *Pythium*-inoculated rice roots provided molecular support for the induced necrosis that accompanied successive accumulation of H_2_O_2_ and phenolic compounds. Analyses of the transcript levels at various times suggested that infections with the *P. arrhenomanes* isolate may have induced a higher degree of necrosis, confirming the higher susceptibility of rice seedlings to this pathogen [8], while the induction of necrosis might have been delayed or weaker upon inoculation with the *P. inflatum* isolate.

The activation of this jasmonic acid (JA)-responsive gene also implies a role for the JA response in the *Pythium*-rice interaction. Many papers have reported on the defense-inducing role of jasmonates (JAs) in *Pythium*-dicot interactions [10,19,24]. In rice, however, salicylic acid (SA) and gibberellic acid (GA) pathways have been identified as regulators of root defense to *Pythium* spp. [4]. The latter research also revealed the ability of *P. graminicola* to hijack the brassinosteroid (BR) machinery in rice seedling roots and hence, negate GA- and SA-dependent immune responses. This seemed to be paralleled with the indirect stabilization of the GA-repressor SLENDER RICE1 (SLR1), i.e. the rice DELLA protein. Interestingly, a recent paper by Yang et al. (2012) [25] reported on the antagonism between JA and GA in rice, and described the DELLA-stabilizing effect of JA. Based on these findings, we put forth a possible model in which *Pythium* infection directly or indirectly activates the JA pathway in rice, by which SLR1 is stabilized and roots become more susceptible. A similar negative role for JA has only been detected in *Fusarium oxysporum*-Arabidopsis interactions, where the pathogen hijacks COI-mediated JA signaling to promote disease development [26]. More research is required to verify this hypothesis and uncover the role of JA in the *Pythium*-rice pathosystem.

Since the virulence and root colonization capacity of rice-infecting *Pythium* spp. seems to be positively linked, we compared the nutritional profiles of the *P. arrhenomanes*, *P. graminicola* and *P. inflatum* isolates to elucidate whether highly virulent species could be physiologically more adapted to their colonization niche or could use specific nutrients as a defense strategy. It was interesting to see that the nutritional profiles of the highly virulent *P. arrhenomanes* and moderately virulent *P. graminicola* isolates were quite similar and clearly different from that of the weakly virulent *P. inflatum* isolate. Carbon-utilization patterns proposed *P. inflatum* as nutritionally the most versatile species. Similar findings were obtained from comparative analyses between plant pathogenic and non-pathogenic *Pseudomonas* species, in which pathogenic species exhibited reduced nutritional versatility [27].

Especially the carbohydrate-utilization profile of the *P. inflatum* isolate was more extensive than that of the *P. arrhenomanes* and *P. graminicola* isolate. Nevertheless, the three *Pythium* isolates grew very well on sucrose, maltose, gentiobiose, D-cellobiose and α-D-glucose and furthermore, *P. arrhenomanes* and *P. graminicola* isolates strongly multiplied on the storage polysaccharide glycogen, and D-trehalose and D-fructose, respectively. These carbohydrates were among those previously mentioned as growth-stimulators of *P. aphanidermatum*[28], *P. myriotylum*, *P. dissotocum*, *P. arrhenomanes*[29] and *P. oligandrum*[30], and which represent, together with other carbohydrates, the largest part of the rice rhizodeposition [31]. In addition, *P. inflatum* and *P. arrhenomanes* isolates were able to grow on D-galactose, which is abundantly exuded by rice roots [32]. Hence, we hypothesize that especially *P. inflatum* and *P. arrhenomanes* and to a lesser extent *P. graminicola* might be physiologically adapted to use carbohydrates likely to be present in the rice rhizosphere. This may also explain the stronger superficial colonization of rice tissues by the *P. arrhenomanes* and *P. inflatum* isolates during our *in vitro* analysis.

The tested *P. inflatum* isolate also exhibited the broadest carboxylic acid-utilization pattern. Malic acid, citric acid and formic acid are known to be present in the phloem sap of rice plants [33] and because our data illustrated that *P. inflatum*, *P. arrhenomanes* and/or *P. graminicola* isolates could grow on these carbon sources, they might serve as nutrient sources inside the rice root stele. The intracellular uptake of these nutrients might have proceeded via haustoria-like structures, which have been described for *P. irregulare* on Arabidopsis [10].

In contrast to the *Pseudomonas* study where plant pathogenic species showed to be specialized in the use of the six most abundant amino acids in their primary infection site [27], we noted that the most virulent *Pythium* isolate exhibited the broadest amino acid-utilization pattern. All *Pythium* isolates were able to grow on a consistent group of nine amino acids that have been proven to attract *Pythium* zoospores [34], stimulate the zoospore encysting process [34] and/or promote the growth of various *Pythium* spp. [28-30].

Among these, especially histidine, proline and alanine are exuded by rice seedlings during the first weeks after planting [32], and asparagine and glutamate are present in the rice phloem [35]. Accordingly, these carbon sources may be used during the outer and inner root colonization processes of the *P. arrhenomanes, P. graminicola* and/or *P. inflatum* isolates in our *in vitro* experiments. It was interesting to see that the amino acid-profile of the *P. arrhenomanes* isolate also consisted of D-serine and D-alanine. Up to date, no *Pythium* spp. have been documented to grow on D-enantiomers of amino acids. Since rice seeds contain substantial amounts of D-serine [36], and D-alanine peptides are present in rice tissues [37], this exceptional feature may contribute to the massive colonization of rice seeds and seedling roots by the *P. arrhenomanes* isolate.

The selective utilization of amino acids is not always a nutritional preference. It might also represent a virulence strategy through which pathogens try to lower defense-related compounds [38]. Hydroxyl-L-proline and L-threonine are building blocks of hydroxyproline-rich glycoproteins (also called extensins), which are present in the primary cell wall of monocots [39] and are functionally implicated in cell wall extension and peroxidase-mediated cell wall fortification [39]. The screened isolates of *P. arrhenomanes* and *P. graminicola* showed to grow on respectively both amino acids and hydroxyl-L-proline only, which may suggest that *P. arrhenomanes* has a stronger potential to block cell wall strengthening in rice root tissues. However, our histopathological study did not evidence the inhibition of cell wall fortification events during *Pythium-*infections of rice seedlings, but their occurrence could have been delayed. Aside from this, L-threonine represents one of the dominant free amino acids in the rice phloem sap [35] and may exert growth-suppressive effects during the interaction of plants with obligate biotrophic oomycetes [40]. It might be possible that *P. arrhenomanes* attempts to remove L-threonine from its host tissues to increase its fitness, but this hypothesis needs further investigation.

In our phenoarrays, we also noted that the three *Pythium* isolates used the defense-related amino acid L-proline [41,42]. Additionally, the *P. inflatum* isolate seemed to grow on putrescine, a stress-related polyamine. Since the weakest virulent isolate could grow on both carbon sources, their removal from the environment is probably not determining for the aggressiveness level of rice-pathogenic *Pythium* spp.

## Conclusions

The degree by which *Pythium* spp. can feed on amino acids, and invade rice cortical and stelar cells seems to be related with the intensity of *Pythium*-induced stunting and wilting symptoms in rice seedlings. Our data suggest that highly virulent *Pythium* species quickly and massively colonize rice root tissues, probably by suppressing cell-wall fortification events, removing defense-related compound from rice tissues, and growing on D-amino acids of which rice seeds contain substantial amounts. Quick invasion of the vascular stele might be of utmost importance for the virulence level of rice-infecting *Pythium* spp., since the rice root endodermis and vascular tissues become suberized/lignified during maturation [43]. This could explain why rice seedlings acquire a certain degree of *Pythium*-resistance within eight days after planting [44]. Interestingly, gibberellins have been noted to mediate lignification of monocot roots [45]. Since GA induces *Pythium*-resistance in rice seedlings, this might reveal why pathogenic *Pythium* spp. try to suppress the GA pathway by inducing the counteracting BR-pathway in rice seedling roots [4]. In this way, they may counterbalance or postpone cell wall fortification in rice seedling roots, i.e. the most important defense reactions against oomycetes.

## Methods

### *Pythium* isolates

In this study, we selected one isolate each of *P. arrhenomanes*, *P. graminicola* and *P. inflatum* to study the interaction between *Pythium* and rice (Table 4). These isolates exhibited different levels of virulence under *in vitro* conditions [3]. Isolates were cultured on 22 ml of potato dextrose agar (PDA; Difco Laboratories) at 28°C in the dark.

**Table 4:** **Origin of the *Pythium *** **strains that were used in this study**

**Species**	**Isolate**	**Geographic origin**	**Year of isolation**	**Collection no.**^**a**^
*P.arrhenomanes*	PT 60	The Philippines, Tarlac (Dapdap)	2007	MUCL52737
*P. graminicola*	PB912 132	The Philippines, Los Baños (IRRI)	2008	MUCL53742
*P. inflatum*	PT 52	The Philippines, Tarlac (Dapdap)	2007	MUCL53750

^a^All isolates were recovered from the roots of aerobic rice plants [3].

### Plant material and infection trials

The rice cultivar Nipponbare (*O. sativa* subspecies *japonica*) was selected for the *in vitro* monitoring of disease symptoms caused by *P. arrhenomanes*, *P. graminicola* and *P. inflatum* on rice roots and shoots. Seeds were as susceptible to *Pythium* as the aerobic rice cv. ‘Apo’, the original host. Prior to germination, seeds were disinfected by agitation in 70% EtOH (1 min) and 2% NaOCl (15 min). After three successive rinses in sterile demineralized water and blotting on sterile filter paper, surface-sterilized seeds were plated on square Petri dishes (120 × 120 mm) filled with 50 ml of Gamborg B5 (GB5) medium [46]. Plates were incubated at 28°C in the dark for three days. Subsequently, seedlings with equal primary root lengths (1.5 cm) were selected and 6 seedlings were transplanted 2 cm apart on fresh GB5 plates. Three mycelial plugs (5 mm in diameter) taken from the edge of a three-day-old *P. arrhenomanes, P. graminicola* or *P. inflatum* colony were placed between the roots of seedlings 1–2, 3–4 and 5–6. Square Petri dishes were partly covered with aluminum foil to shield the roots from light and then, incubated in upright position (60°C angle) inside a growth chamber with a 12 h day (28°C) / night (26°C) cycle. The experiment consisted of three replications per treatment.

**Figure 9:**
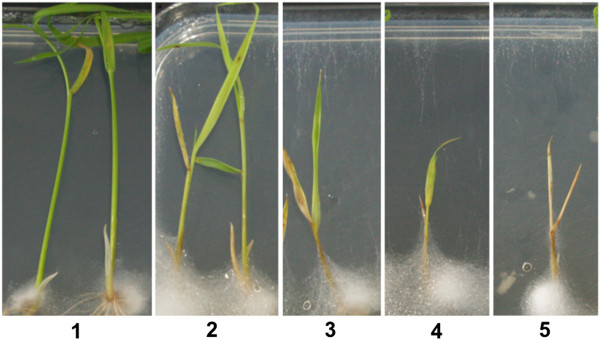
**The disease severity scale implemented for disease rating on rice shoots.** Shoot disease severity scores (1-5) are based on the degree of stunting and wilting.

For disease evaluation, maximal root and shoot lengths were measured ten days post inoculation (dpi) (n = 18). Disease symptoms on rice shoots were rated on the basis of a disease severity scale (Figure 9) (n = 3): score 0, healthy shoots; score 1, shoot length ≥ 50% of the control, green culm and few yellow or brown spotted leaves; score 2, shoot length ≥ 34% of the control, slightly yellowing culm and yellow or brown spotted leaves; score 3, shoot length < 34% of the control, slightly yellowing culm and yellow or brown spotted leaves; score 4, shoot length < 34% of the control, yellow culm and yellow or brown leaves; score 5, shoot length < 34% of the control, brown, dried-out culm and leaves. This scale enabled us to calculate the disease severity index (DSI) for each biological replicate (i.e. Petri dish) using the following equation: (((# × score 0) + (# × score 1) + (# × score 2) + (# × score 3) + (# × score 4) + (# × score 5)) / ((total #) × score 5))) × 100 (with # the no. of seedlings). Most data were not normally distributed and statistically analyzed with Kruskal-Wallis and Mann-Whitney non-parametric tests in SPSS 21 (SPSS Inc.) (α = 0.05, *P* ≤ α). Data that were normally distributed were analyzed by one-way ANOVA and a Duncan Post-Hoc test (α = 0.05, *P* ≤ α).

### Microscopic analysis of root samples

Root infection processes were analyzed at various times after *P. arrhenomanes, P. graminicola* and *P. inflatum* inoculation using bright field and epifluorescence microscopy. In this experiment, GB5-culture plates contained four rice seedlings next to which four mycelial plugs were placed at 0.5 cm distance at the right side of each emerging radicle. Parts of the primary roots showing superficial hyphal growth and necrosis were excised from *Pythium*-inoculated rice seedlings. Root samples of ± 0.5 cm were fixated in a 50 mM sodium phosphate buffer (pH 7.2) containing 4% paraformaldehyde and 1% glutaraldehyde, and subsequently dehydrated in a graded series of EtOH and infiltrated with Technovit 7100 solution.

The infiltrated root samples were finally embedded in plastic 1 cm^2^ - cubes filled with Technovit 7100 histo-embedding medium (Heraeus Kulzer, Wehrheim, Germany). A Leica RM2265 motorized rotary microtome (Leica Microsystems, Nussloch, Germany) was used to produce 5 μm cross-sections. For each treatment at different time points, three to four root samples of at least two different seedlings were entirely sectioned. This resulted into 50 cuttings per sample, which were randomized on ten microscopic slides. At least two slides were chosen for each staining procedure. *Pythium* hyphae were stained by incubation of sections in 0.1% (w/v) trypan blue in 10% (v/v) acetic acid for 5 min. The accumulation of phenolic compounds was visualized by staining with 0.1% Fluorescent brightener 28 (Calcofluor White M2R) for 1 min. All stained sections were thoroughly rinsed, dried and mounted with neutral mounting medium (DPX, Klinipath, Belgium). The accumulation of hydrogen peroxide (H_2_O_2_), a marker for plant defense and induced necrosis, was demonstrated by staining of fresh root samples with 3,3’-diaminobenzidine (DAB). Three to four root samples were collected from at least two different seedlings for each treatment at various time points. Immediately after sampling, vacuum-infiltration with a 0.1% 3,3’-diaminobenzidine (DAB)-solution (pH 4.4) was performed in three successive steps of 5 min in the dark. After an extra incubation step of 4 min, the residual stain was removed and the samples were fixated, sectioned and mounted as described above. The DAB-stained specimens were also treated with trypan blue to visualize *Pythium* hyphae. Digital images were acquired with an Olympus BX51 microscope equipped with an Olympus ColorView III Camera and Xenon light source. A DAPI narrow-band fluorescence cube (BP330-385 nm/DM400/BA420) was selected for analyses with the fluorescent stain. Images were processed with the Olympus analysis cell^F software (Olympus Soft Imaging Solutions, Münster, Germany) and ImageJ 1.44p.

### Quantitative detection of *Pythium* spp. in root samples

Primers specific for the multi-copy Internal Transcribed Spacer (ITS)-region of the ribosomal DNA (rDNA) of *P. arrhenomanes* PT 60, *P. graminicola* PB912 132 and *P. inflatum* PT 52 were constructed using Primer-BLAST (NCBI) and OligoAnalyzer 2.1. web-software (IDT, Coralville, IA) (Table 5). The species specificity of the primers was double-checked with pure DNA of the three *Pythium* spp. and rice root DNA (cv. Nipponbare). Optimal annealing temperatures were assessed by gradient PCR on a thermal cycler (Flexcycler, Analytikjena) and a primer titration was executed on a Mx3005P real-time PCR detection system (Stratagene) using Sybr Green master mix (Fermentas). After setting the optimal reaction conditions, standard curves based on quinquepartite dilution series (10 ng - 1 pg) were run on the real-time PCR to monitor the amplification efficiency and accuracy of the primer pairs. If the curves showed R^2^ ≥ 0.985, slopes between −3.1 à −3.6 and efficiencies between 90-110%, primers were accepted.

**Table 5:** Sequences of the species-specific primers that amplify a part of the ITS1 region

***Pythium*** **isolate**	**Forward ITS primer (5′-3′)**^**a**^	**Reverse ITS primer (5′-3′)**^**a**^	**Size amplicon**	**GenBank Accession no.**^**b**^
*P. arrhenomanes* PT 60	ATTCTGTACGCGTGGTCTTCCG (3 μM)	ACCTCACATCTGCCATCTCTCTCC (1 μM)	311 bp	HQ877857
*P. graminicola* PB912 132	ATGGCTGAACGAAGGTGGGCTG (1 μM)	TCCCGAAAGTGCAATGTGCGTTC (3 μM)	240 bp	HQ877865
*P. inflatum* PT 52	AGGTGGGCGCATGTATGTGTGTC (500 nM)	ACGTATCGCAGTTCGCAGCG (3 μM)	165 bp	HQ877856

^a^The concentrations for each primer stock are presented between brackets.

^b^The ITS-regions of *P. arrhenomanes* PB912 75 (HQ877857) and *P. graminicola* PB912 116 (HQ877865) are respectively identical to those of *P. arrhenomanes* PT 60 and *P. graminicola* PB912 132 [3].

*Pythium*-inoculated rice roots were collected at different time points from rice-seedling culture plates, in two separate experiments (see “Plant material and infection trials”). In each experiment, samples from two replicate plates were pooled per treatment at each time point (n = 2). Samples were immediately frozen in liquid nitrogen or prior to freezing surface-sterilized in 1% NaOCl for 1 min and thoroughly washed. Next, DNA was extracted from the finely crushed roots with the DNeasy Plant Mini Kit (QIAGEN). The quality and concentration of the extracted DNA was determined with a ND-1000 spectrophotometer (NanoDrop). If necessary, ethanol precipitation was applied to concentrate the DNA sample. The extracted DNA (2.5 μl of 1 ng/μl) was added to 96-well plates, filled with 12.5 μl aliquots of Sybr Green master mix (Fermentas) that were supplemented with 2.5 μl of each primer stock solution (Table 2), 0.05 μl of ROX solution and 4.95 μl of nuclease-free water. Each DNA sample was analyzed in duplicate with the Mx3005P real-time PCR detection system (Stratagene) using the following thermal profile: an initial denaturation at 95°C for 10 min, and 40 cycles of 15 s at 95°C, 30 s at 63°C and 15 s at 72°C. To verify amplicon specificity, a default melting-curve analysis (Stratagene) was included. Finally, cycle threshold (Ct) values (n = 4) were implemented in the standard curves’ equations to quantify the amount of *Pythium* DNA in the collected root samples.

### Gene expression analysis in *Pythium*-inoculated rice roots

Rice roots were collected from GB5-culture plates at different times upon *Pythium* inoculation (see “Plant material and infection trials”). In each of three experiments, root samples from two replicate plates were pooled per treatment at each time point (n = 3). Samples were frozen in liquid nitrogen, finely crushed and afterwards, total RNA was extracted using the spectrum plant total RNA kit (Sigma-Aldrich). A Turbo Dnase treatment (Ambion) was immediately performed and the quality and concentration of the extracted RNA was determined with a ND-1000 spectrophotometer (NanoDrop). Next, complement DNA (cDNA) was synthesized from the total RNA (10 ng/μl) with Multiscribe reverse transcriptase and random primers (Applied Biosystems). This cDNA (2.5 μl of 10 ng/μl) was added to 96-well plates, filled with 12.5 μl aliquots of Sybr Green master mix (Fermentas) that were supplemented with 2.5 μl of each primer stock solution (Table 3), 0.05 μl of ROX solution and 4.95 μl of nuclease-free water. Each sample was analyzed in duplicate with the Mx3005P real-time PCR detection system (Stratagene) under the following conditions: an initial denaturation step at 95°C for 10 min, and 40 cycles of 15 s at 95°C, 30 s at 59°C and 15 s at 72°C. After the PCR, a default melting curve (Stratagene) was generated to test amplicon specificity. The quantity of plant RNA in each sample was normalized using *OsACTIN1* (LOC_Os03g50890) as internal reference (Table 6). Ct values were relatively expressed to the non-inoculated control at 1 dpi and the average fold change of the three experiments (n = 3) was calculated.

**Table 6:** Sequences of the used primers for gene expression analysis

**Gene**	**Forward primer (5′-3′)**^**a**^	**Reverse primer (5′-3′)**^**a**^	**GenBank Accession no.**
*OsJAmyb*	GAGGACCAGAGTGCAAAAGC (3 μM)	CATGGCATCCTTGAACCTCT (3 μM)	AY026332
*OsACTIN1*	GCGTGGACAAAGTTTTCAACCG (1 μM)	TCTGGTACCCTCATCAGGCATC (3 μM)	X15865

^a^The concentrations for each primer stock are presented between brackets.

### Phenoarrays

The carbon-utilization patterns of pure *P. arrhenomanes, P. graminicola* and *P. inflatum* cultures were investigated *in vitro* with SF-N2 and SF-P2 MicroPlates (Biolog Inc.) based on the protocol of Chun et al. (2003) [30]. In these MicroPlates, each well contains a nutrient base with specific carbon sources, including several carbohydrates, amino acids and carboxylic acids. *Pythium* strains were cultured in 50 ml of potato dextrose broth (PDB; Difco Laboratories) at 28°C in the dark. After 11 days, liquid cultures were filtered with sterile sieving cloth (1 mm mesh) and the retained mycelial mats were washed in three rinses with sterile demineralized water. The harvested mycelium was subsequently mixed in a sterile 1 mM potassium phosphate buffer (pH = 7) and again sieved (1 mm mesh). Optical densities of the suspensions were determined at 595 nm in triplicate using a Multiscan EX spectrophotometer (Thermo Labsystems). Next, OD’s were adjusted to 0.100 ± 0.006 by dilution in sterile phosphate buffer, and three SF-N2 and three SF-P2 MicroPlates were filled with 100 μl aliquots of each mycelial suspension. Control plates were filled with 100 μl aliquots of sterile phosphate buffer. Upon an incubation step of 24 h at 28°C in the dark, carbon source utilization patterns of *P. arrhenomanes* PT 60, *P. graminicola* PB912 132 and *P. inflatum* PT 52 were assessed by turbidity measurements at 595 nm. The experiment consisted of three replicate plates and was repeated in time to verify the reproducibility. This generated at least six OD-values per carbon source for each treatment (n ≥ 6). Data were statistically analyzed using Kruskal-Wallis in SPSS 21 (SPSS Inc.) (α = 0.05, *P* ≤ α). When turbidities significantly exceeded the initial OD (= 0.1), *Pythium* spp. were assumed to use the according carbon sources for their growth.

## Competing interests

The authors declare that they have no competing interests.

## Authors’ contributions

VBE carried out the experiments, interpreted the data, drafted and edited the manuscript. HM coordinated the interpretation of data and drafting, revised the manuscript critically and gave final approval for publication. Both authors read and approved the final manuscript.

## Supplementary Material

Additional file 1: Figure S1 The stimulating effect of rice seed exudates on *Pythium* growth. Rice seeds (2.5 g) of the cv. CO-39 (*O. sativa* subspecies *indica*), which is as susceptible to *Pythium* as cvs. Apo and Nipponbare, were surface sterilized, washed and incubated in 20 ml of sterile demineralized water at 28°C. Seed exudates were collected as a watery solution after 24 h of imbibition. Three ml aliquots of water (−) or exudate solutions (+) were added to three six-well replicate plates and afterwards, inoculated with one PDA plug of a four-day old *P. arrhenomanes, P. graminicola* or *P. inflatum* culture. Plates were incubated at 28°C and screened after 17 h. A clear stimulation in colony diameter and/or density was visible when *Pythium* spp. were grown in seed exudates. The picture is representative for the three replicate plates. Click here for file

Additional file 2: Table S1 Carbon sources that stimulated *Pythium* growth in the phenoarray. Data represent the carbon sources for which the OD-values significantly differed from the initial OD (= 0.1) at 24 hpi according to Kruskal-Wallis non-parametric tests in SPSS 21 (α = 0.05, *P* ≤ α). Values between brackets represent standard errors. Click here for file

Additional file 3: Table S2 Carbon sources that did not stimulate growth of *Pythium* in the phenoarray. The OD-values of the listed carbon sources did not significantly differ from the initial OD (= 0.1) at 24 hpi according to Kruskal-Wallis non-parametric tests in SPSS 21 (α = 0.05, *P* ≤ α). Click here for file
