# Supplementary figures and images for: Pythium species from rice roots differ in virulence, host colonization and nutritional profile
Source: BMC Plant Biol. 2013 Dec 5;13:203. doi: 10.1186/1471-2229-13-203 (PMC3878986; doi:10.1186/1471-2229-13-203)

*P. arrhenomanes*

*P. graminicola*

*P. inflatum*

+

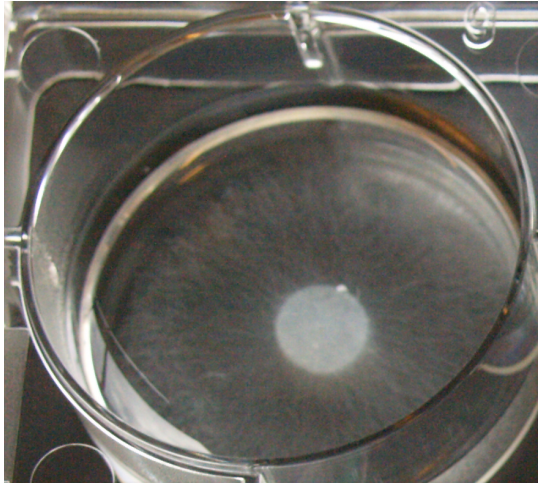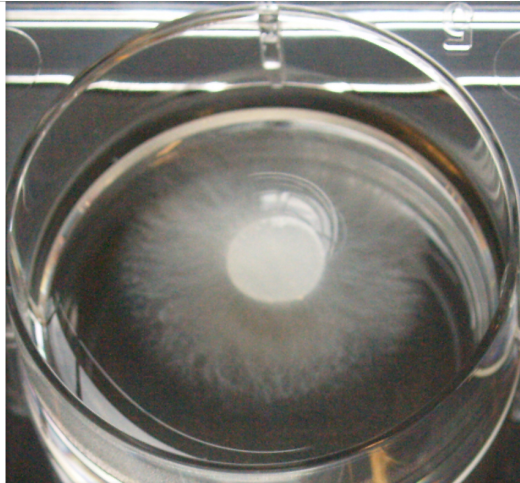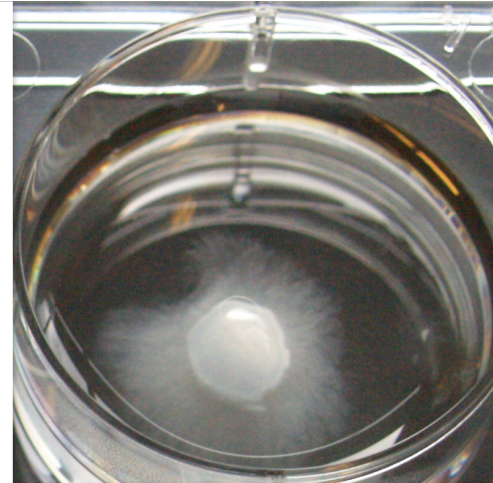

-

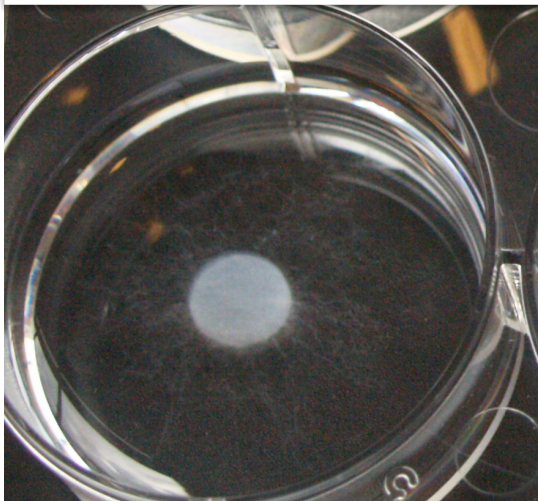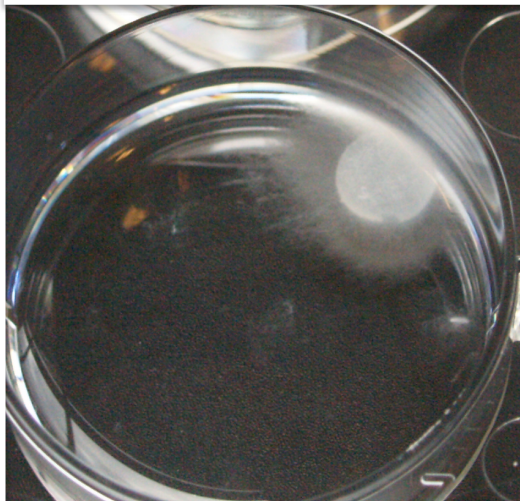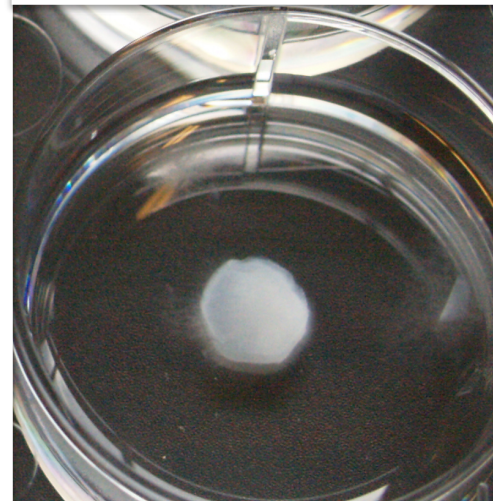

Supplement: Additional file 1: Figure S1 — The stimulating effect of rice seed exudates on Pythium growth. Rice seeds (2.5 g) of the cv. CO-39 (O. sativa subspecies indica), which is as susceptible to Pythium as cvs. Apo and Nipponbare, were surface sterilized, washed and incubated in 20 ml of sterile demineralized water at 28°C. Seed exudates were collected as a watery solution after 24 h of imbibition. Three ml aliquots of water (−) or exudate solutions (+) were added to three six-well replicate plates and afterwards, inoculated with one PDA plug of a four-day old P. arrhenomanes, P. graminicola or P. inflatum culture. Plates were incubated at 28°C and screened after 17 h. A clear stimulation in colony diameter and/or density was visible when Pythium spp. were grown in seed exudates. The picture is representative for the three replicate plates. [file 1471-2229-13-203-S1.pdf]
