# Supplementary material for: Pythium species from rice roots differ in virulence, host colonization and nutritional profile
Source: BMC Plant Biol. 2013 Dec 5;13:203. doi: 10.1186/1471-2229-13-203 (PMC3878986; doi:10.1186/1471-2229-13-203)
Supplement: Additional file 2: Table S1 — Carbon sources that stimulated Pythium growth in the phenoarray. Data represent the carbon sources for which the OD-values significantly differed from the initial OD (= 0.1) at 24 hpi according to Kruskal-Wallis non-parametric tests in SPSS 21 (α = 0.05, P ≤ α). Values between brackets represent standard errors. [file 1471-2229-13-203-S2.doc]

| **C-source** | **Name** | ***P. inflatum*** | ***P. arrhenomanes*** | ***P. graminicola*** |
| --- | --- | --- | --- | --- |
| Amino acid | D-alanine | 0 | 0.22 (0.02) | 0 |
|  | D-serine | 0 | 0.24 (0.01) | 0 |
|  | L-alanine | 0.31 (0.03) | 0.27 (0.03) | 0.26 (0.02) |
|  | L-asparagine | 0.39 (0.02) | 0.30 (0.03) | 0.31 (0.03) |
|  | L-aspartic acid | 0.37 (0.02) | 0.32 (0.02) | 0.28 (0.02) |
|  | L-glutamic acid | 0.33 (0.02) | 0.30 (0.03) | 0.29 (0.02) |
|  | L-histidine | 0.31 (0.01) | 0.27 (0.02) | 0.27 (0.01) |
|  | L-ornithine | 0.28 (0) | 0.29 (0.02) | 0.24 (0.01) |
|  | L-phenylalanine | 0.23 (0.02) | 0.26 (0.01) | 0.23 (0.02) |
|  | L-proline | 0.33 (0.01) | 0.28 (0.01) | 0.25 (0.02) |
|  | L-serine | 0.23 (0.01) | 0.22 (0.02) | 0 |
|  | L-threonine | 0 | 0.22 (0.01) | 0 |
| Amino acid derivate | L-alanyl glycine | 0.28 (0.01) | 0.27 (0.02) | 0.22 (0.02) |
|  | glycyl-L-glutamic acid | 0.24 (0.03) | 0.28 (0.03) | 0.22 (0.01) |
|  | hydroxy-L-proline | 0 | 0.23 (0.02) | 0.21 (0.01) |
|  | L-pyroglutamic acid | 0.20 (0.01) | 0 | 0 |
|  | 2-aminoethanol | 0.23 (0.02) | 0.28 (0.01) | 0.21 (0.01) |
|  | N-acetyl-D-galactosamine | 0.21 (0.02) | 0.25 (0.01) | 0 |
|  | N-acetyl-D-glucosamine | 0.22 (0.03) | 0 | 0 |
|  |  |  |  |  |
|  |  |  |  |  |
| Carboxylic acid | bromosuccinic acid | 0.26 (0.02) | 0 | 0.22 (0.01) |
|  | cis-aconitic acid | 0.24 (0.02) | 0.22 (0.01) | 0.25 (0.01) |
|  | citric acid | 0.27 (0.05) | 0.22 (0.01) | 0 |
|  | D-gluconic acid | 0.23 (0.01) | 0.22 (0.01) | 0.20 (0.01) |
|  | D-glucosaminic acid | 0.21 (0.02) | 0.22 (0.02) | 0.21 (0.02) |
|  | D-glucuronic acid | 0.22 (0.01) | 0 | 0 |
|  | D-malic acid | 0.21 (0.01) | 0 | 0 |
|  | D-saccharic acid | 0.23 (0.02) | 0 | 0 |
|  | D,L-lactic acid | 0.32 (0.01) | 0.31 (0.01) | 0.22 (0.02) |
|  | formic acid | 0 | 0.25 (0.01) | 0.20 (0.01) |
|  | itaconic acid | 0 | 0.23 (0.01) | 0 |
|  | L-malic acid | 0.27 (0.02) | 0 | 0 |
|  | malonic acid | 0.23 (0.01) | 0.22 (0) | 0.20 (0.01) |
|  | quinic acid | 0.22 (0.01) | 0 | 0 |
|  | succinamic acid | 0.21 (0.01) | 0.22 (0.02) | 0 |
|  | succinic acid | 0.22 (0.01) | 0.25 (0.03) | 0.25 (0.03) |
|  | α-ketobutyric acid | 0 | 0.25 (0.01) | 0.21 (0.01) |
|  | D-galacturonic acid | 0.23 (0.01) | 0.23 (0.01) | 0.19 (0.01) |
| Carboxylic acid derivate | L-alaninamide | 0.28 (0.02) | 0.25 (0.03) | 0.22 (0.02) |
|  | glucuronamide | 0.22 (0.01) | 0 | 0 |
|  | D-galactonic acid lactone | 0.23 (0.01) | 0.24 (0.02) | 0 |
|  | α-ketoglutaric acid | 0.23 (0.02) | 0.28 (0.02) | 0.23 (0.02) |
|  | pyruvic acid | 0.21 (0.01) | 0.22 (0.01) | 0 |
|  | pyruvic acid methyl ester | 0 | 0.24 (0.03) | 0 |
|  | succinic acid mono-methyl ester | 0.21 (0.01) | 0 | 0 |
|  | β-hydroxybutyric acid | 0.20 (0.01) | 0 | 0 |
|  |  |  |  |  |
|  |  |  |  |  |
| Carbohydrate | D-cellobiose | 0.46 (0.07) | 0.40 (0.07) | 0.42 (0.03) |
|  | D-fructose | 0.29 (0.02) | 0.33 (0.03) | 0.42 (0.02) |
|  | D-galactose | 0.24 (0.01) | 0 | 0 |
|  | D-mannose | 0.32 (0.05) | 0.23 (0.02) | 0.21 (0.08) |
|  | D-melezitose | 0.21 (0.01) | 0 | 0 |
|  | D-melibiose | 0.21 (0.01) | 0 | 0 |
|  | D-psicose | 0.23 (0.01) | 0 | 0 |
|  | D-raffinose | 0.22 (0.01) | 0.26 (0.02) | 0.22 (0.01) |
|  | D-ribose | 0.22 (0.01) | 0 | 0 |
|  | D-trehalose | 0.31 (0.02) | 0.27 (0.02) | 0.41 (0.03) |
|  | dextrin | 0.32 (0.04) | 0.32 (0.04) | 0.33 (0.02) |
|  | gentiobiose | 0.46 (0.06) | 0.38 (0.05) | 0.35 (0.03) |
|  | glycogen | 0.32 (0.04) | 0.37 (0.05) | 0.29 (0.03) |
|  | L-arabinose | 0.22 (0.02) | 0 | 0 |
|  | L-fucose | 0.23 (0.01) | 0.22 (0.01) | 0.20 (0.01) |
|  | L-rhamnose | 0.23 (0.01) | 0.23 (0.01) | 0.19 (0.01) |
|  | lactulose | 0.27 (0.02) | 0 | 0.19 (0.01) |
|  | maltose | 0.48 (0.06) | 0.39 (0.04) | 0.41 (0.04) |
|  | maltotriose | 0.31 (0.01) | 0.27 (0.02) | 0.32 (0.04) |
|  | mannan | 0.22 (0.01) | 0 | 0 |
|  | palatinose | 0.23 (0.01) | 0 | 0 |
|  | sucrose | 0.46 (0.08) | 0.43 (0.05) | 0.41 (0.03) |
|  | turanose | 0.22 (0.01) | 0 | 0 |
|  | α-D-glucose | 0.56 (0.07) | 0.47 (0.04) | 0.48 (0.02) |
|  | α-D-lactose | 0.26 (0.03) | 0.23 (0.02) | 0.24 (0.02) |
| Carbohydrate derivate | arbutin | 0.27 (0.01) | 0.26 (0.01) | 0.26 (0.02) |
|  | salicin | 0.26 (0.01) | 0.23 (0.02) | 0.25 (0.03) |
|  | α-methyl-D-galactoside | 0.21 (0.01) | 0 | 0 |
|  | α-methyl-D-mannoside | 0.23 (0.01) | 0 | 0 |
|  | β-methyl-D-galactoside | 0.24 (0.01) | 0 | 0 |
|  | β-methyl-D-glucoside | 0.38 (0.06) | 0.28 (0.03) | 0.24 (0.02) |
|  | 2,3-butanediol | 0 | 0.22 (0.01) | 0 |
|  | adonitol | 0.24 (0.01) | 0 | 0.20 (0.01) |
|  | D-arabitol | 0.23 (0.02) | 0 | 0.20 (0.01) |
|  | D-mannitol | 0.22 (0.01) | 0.22 (0.01) | 0 |
|  | D-sorbitol | 0.21 (0.01) | 0 | 0 |
|  | glycerol | 0.36 (0.05) | 0.25 (0.03) | 0.28 (0.03) |
|  | i-erythritol | 0.21 (0.01) | 0 | 0 |
|  | m-inositol | 0.24 (0.02) | 0.22 (0.01) | 0.22 (0.01) |
|  | xylitol | 0.22 (0.01) | 0 | 0 |
|  |  |  |  |  |
|  |  |  |  |  |
| Amine | phenylethylamine | 0.25 (0.02) | 0.26 (0.02) | 0 |
|  | putrescine | 0.20 (0.01) | 0 | 0 |
| Nucleoside | inosine | 0 | 0.22 (0.01) | 0 |
|  | thymidine | 0 | 0.21 (0.01) | 0 |
|  | uridine | 0 | 0.22 (0.02) | 0 |
| Surfactants | tween 40 | 0.43 (0.03) | 0.42 (0.04) | 0.43 (0.03) |
|  | tween 80 | 0.37 (0.05) | 0.32 (0.06) | 0.38 (0.04) |
|  |  |  |  |  |
