# Supplementary material for: Pythium species from rice roots differ in virulence, host colonization and nutritional profile
Source: BMC Plant Biol. 2013 Dec 5;13:203. doi: 10.1186/1471-2229-13-203 (PMC3878986; doi:10.1186/1471-2229-13-203)
Supplement: Additional file 3: Table S2 — Carbon sources that did not stimulate growth of Pythium in the phenoarray. The OD-values of the listed carbon sources did not significantly differ from the initial OD (= 0.1) at 24 hpi according to Kruskal-Wallis non-parametric tests in SPSS 21 (α = 0.05, P ≤ α). [file 1471-2229-13-203-S3.doc]

| **Carbon source** |
| --- |
| 2'-deoxy adenosine |
| acetic acid |
| adenosine |
| adenosine-5'-Monophosphate |
| D-fructose-6-phosphate |
| D-glucose-6-phosphate |
| D-lactic acid methyl ester |
| D-tagatose |
| D-xylose |
| D,L,α-glycerol phosphate |
| inulin |
| L-lactic acid |
| L-leucine |
| lactamide |
| p-hydroxyphenylacetic acid |
| propionic acid |
| sedoheptulosan |
| thymidine-5'-monophosphate |
| uridine-5'-monophosphate |
| urocanic acid |
| α-cyclodextrin |
| α-D-glucose-1-phosphate |
| α-hydroxybutyric acid |
| α-ketovaleric acid |
| β-cyclodextrin |
| γ-hydroxybutyric acid |
